# Neural Differentiation of Incorrectly Predicted Memories

**DOI:** 10.3389/fnhum.2018.00278

**Published:** 2018-07-11

**Authors:** Andrea Greve, Hunar Abdulrahman, Richard N. Henson

**Affiliations:** MRC Cognition and Brain Sciences Unit, University of Cambridge, Cambridge, United Kingdom

**Keywords:** neural differentiation, prediction error, neural integration, fMRI, episodic memory, neural network simulation

Frequently experiencing an item in a specific context leads to the prediction that this item will occur when we encounter the same context in future. However, this prediction sometimes turns out to be incorrect, and recent behavioral research suggests that such “prediction errors” improve encoding of new information (Greve et al., 2017). In their recent article “Neural differentiation of incorrectly predicted memories,” Kim et al. (2017) address the interesting question of how neural representations of items change when they are incorrectly predicted and subsequently restudied. The authors conclude such items undergo representational differentiation, i.e., a decreased overlap in the representations of an item and its context. We suggest the reverse mechanism of integration, rather than differentiation, is equally compatible with current evidence. In particular, we consider how Kim et al.'s results fit with recent suggestions about prediction-error driven learning and transitive inference.

In their fMRI study, Kim et al. (2017) use pattern similarity analysis across voxels to assess representational change for incorrectly predicted items at four different phases. The items were visual scenes, randomly split into three types: A, B, and X. A first “pre-study” phase showed a random sequence of these scenes in order to measure their initial neural representations, here called “pre-A,” “pre-B,” and “pre-X” (Figure 1a). In a subsequent “study” phase, scene A was shown three times, always followed by scene B, forming AB pairs (Figure 1b). In a third phase, the “critical study” phase, half of the trials were followed by two cycles of violations (i.e., A was followed by X rather than B) and restudy (presentation of B), while the other half was followed by restudy only (no-violation). In a final “post-study” phase, all B items (post-B) were presented again in random order to measure their representational change.

**Figure 1:**
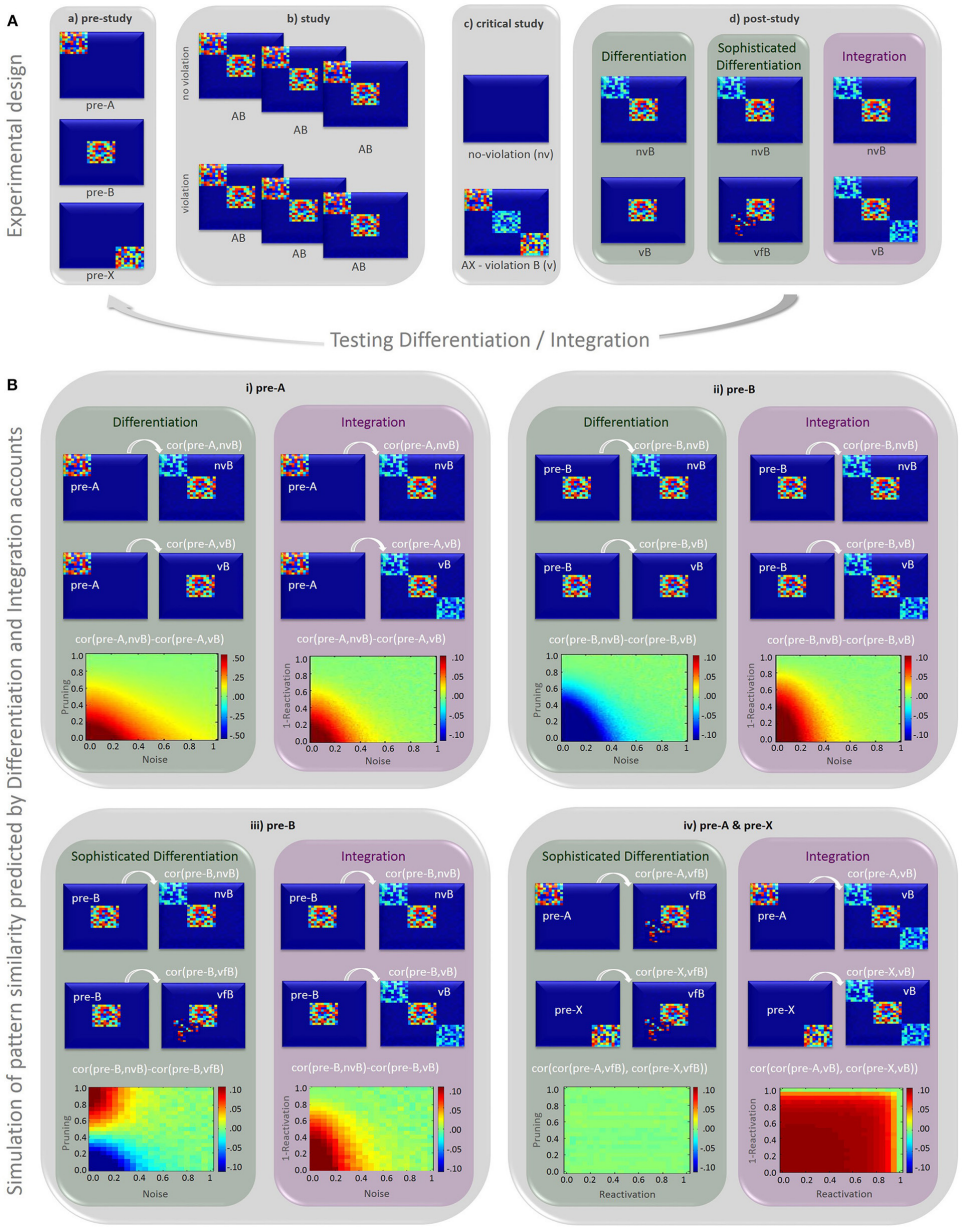
**(A)** Illustration of simulated activation patterns representing scenes and their changes through the different phases in Kim et al.'s experiment. **Panel a** pre-study: initial representation of individual scenes. **Panel b** study: repeated pairing of A and B leads to co-activation of both scene representations. **Panel c** critical study: the co-activation of A and B representation remains unchanged in the no-violation (nv) condition. In the violation (v) condition, the prediction of B (vB) given A leads to moderate re-activation of B representations. The item X causing the prediction error becomes co-activated with A. **Panel d** post-study: in the no-violation (nv) condition, B (nvB) co-activates A representations according to both accounts. In the violation (v) condition, however, pruning of B (vB) from A representations is predicted by the differentiation account (vB, left), with assimilation of some new, unique features predicted by the sophisticated differentiation account (vfB, middle), while moderate re-activation of **(A)** and X representations given **(B)** is expected by the integration account (vB, right). **(B)** scene representations are simulated across a 30 × 30 voxel space. (For simplicity we assume no overlap between item representations in pre-study, but qualitative results remain unchanged if overlap is introduced). Boundary conditions under which the differentiation and integration account predict similar or distinct patterns of results are explored by using different levels of random noise (x-axis) and pruning/re-activation strength (y-axis), implemented using a range of weights (0–1). Each **sub-panel (i–iv)** shows the simulated contrast at the top and the resulting pattern correlation “cor” at the bottom, for the differentiation (green box to the left) and Integration (red box to the right) account. The calculation of correlations is specified in the equation presented in each panel, (code for all these simulations can be found here: https://osf.io/exz5b/). The y-axis in each plot shows different levels of pruning/reactivation of associated items that are not presented, i.e., pattern A and X when pattern B is presented in the post-study condition (the degree of pruning in the differentiation model and reactivation in the Integration model are inversely yoked). The x-axis shows different levels of random noise in the (no)violation and post-study phase (or degree of reactivation in plot iv). **Panel i** confirms that both the differentiation and integration account predict that the correlation between “pre-A” and “no-violation B” (nvB) patterns is greater than that between “pre-A” and “violation B” (vB), which was the result reported by Kim et al. (2017). **Panel ii** however shows that the integration account, but not a simple differentiation account, predicts that the correlation between “pre-B” and “no-violation B” (nvB) patterns is greater than between “pre-B” and “violation B” (vB). **Panel iii** shows the same contrast as Panel ii, but with the addition of new features in the violated B representations (vfB), according to the more sophisticated differentiation account of Kim et al. **Panel iv** shows how the similarity between “pre-X” and “violated B” representations (vfB) is correlated with the similarity between “pre-A” and “vfB,” which is predicted by the integration but not either type of differentiation account.

Based on prior neural network simulations (Norman et al., 2006), the authors predicted post-B items to be more dissimilar to pre-A items in the violation compared to no-violation condition. This prediction is based on the assumption repeated study of AB pairs will strongly co-activate their representations, leading to the prediction of item B when presented with item A (see Figure 1c). If predictions are violated when A is followed by X instead of B, the co-activation of A and B is weakened (i.e., “pruned”) and the representation of B is thought to “differentiate” from the representation of A (Figure 1d, simple differentiation). This process of neural differentiation is an appealing computational mechanism that has been used to explain other findings (e.g., Hulbert and Norman, 2015).

Since post-A representations are confounded by learning history (study frequency and context), Kim et al. assessed “differentiation” by examining the similarity (correlation of patterns across voxels) between post-B and pre-A trials. The logic is AB pairs remain intact in the no-violation condition, so that post-B items bring A items to mind (i.e., “reactivate” A), producing a degree of similarity between post-B and pre-A representations. For the violation condition, however, differentiation reduces the similarity between post-B and pre-A representations. Thus post-B and pre-A patterns should be more highly correlated in the no-violation than violation condition, which was indeed reported by Kim et al, and confirmed by simulations in top plot of Figure 1Bi.

However, we suggest the results are equally compatible with an alternative account: the integration hypothesis, by which the item creating the prediction error (X) becomes associated with item A and the predicted item B, forming an integrated ABX representation (Figure 1d, integration). The additional X representation can produce the same result Kim et al. found, namely higher similarity for no-violations (pre-A vs. post-AB) compared to violations (pre-A vs. post-ABX; bottom plot of Figure 1Bi). Thus, Kim et al.'s findings are equally consistent with a process of integration. Indeed, previous studies show violations of expectations can foster better associative learning between items eliciting a prediction error (Greve et al., 2017), suggesting that A and X in the Kim et al. design could become bound together. Moreover, studies of transitive learning, using a similar design to Kim et al., show facilitated integration (Zeithamova et al., 2012; Schlichting et al., 2015).

Although the analysis reported by Kim et al. cannot dissociate between differentiation and integration, our simulations show that other contrasts reveal opposing predictions for these two accounts. For instance, comparing representations of post-B with pre-B items, instead of pre-A items, predicts higher similarity for non-violation vs. violation conditions according to the integration account, but lower similarity according to the simplistic differentiation account considered so far (Figure 1Bii), across a range of noise and reactivation/pruning levels^1^.

However, Kim et al.'s differentiation account is more sophisticated, and states that “… if B is restudied later … activation will spread to other new features (not previously shared with A) and connections to these features will be strengthened.” This more sophisticated differentiation account has also been used in previous studies, for instance to account for recall of competing memories (Hulbert and Norman, 2015). Here, the inclusion of new features following violation of B (vfB) is illustrated in Figure 1d (sophisticated differentiation), and simulated in Figure 1Biii. In the simulations, it can be seen that under low noise levels and high levels of pruning, this sophisticated differentiation account can predict higher similarity between post-B and pre-B items for non-violation vs. violation conditions, making it impossible to dissociate it from the integration account in their paradigm (since we do not know levels of noise or pruning/reactivation in reality).

Nevertheless, the type of information assimilated can still distinguish the two accounts. One straightforward approach would be to compare pattern similarity between pre-X and post-B items: Higher similarity is predicted in the violation condition according to the integration but not differentiation account. Unfortunately, however, empirical evidence cannot be obtained by Kim et al. (2017) because they did not present pre-X items in the no-violation condition. Another approach, which could be tried on Kim et al.'s data, is to correlate the similarity of post-B items and pre-A items in the violated condition with the similarity between post-B items and pre-X items in the violated condition: according to the integration account, there should be a positive correlation, because stronger AB associations should evoke larger prediction errors and hence stronger associations between X and B. Since X is not part of the post-B representation according to the differentiation account, no such correlation is predicted (Figure 1Biv)^2^.

In sum, Kim et al. present interesting data that address important questions about how incorrect predictions change neural representations. While we do not think the results reported can yet distinguish a differentiation account from an integration account, our simulations suggest additional comparisons that would help in future.

## Author contributions

AG, HA, and RH contributed to the conceptual argument developed in the commentary. AG and HA simulated the data. RH provided advice how best to simulate and interpret the results. AG wrote the first draft of the manuscript. HA and RH wrote and re-wrote sections of the manuscript. All authors contributed to manuscript revision, read and approved the submitted version.

### Conflict of interest statement

The authors declare that the research was conducted in the absence of any commercial or financial relationships that could be construed as a potential conflict of interest.

## Footnotes

^1^Noise is introduced by adding uniformly distributed random patterns to item representation, using a range of weights (0–1) to simulate different levels of noise. Pruning refers to the weakening of representations for incorrectly predicted items: for example, the features of A are pruned from B, after A is unexpectedly paired with X instead of B. Pruning is implemented by subtracting A from AB representations, again using a range of weights (0–1) for different degrees of pruning. Similarly, different degrees of reactivation are simulated by adding A to B representations with a range of weights (0–1). For simplicity pruning (p) and reactivation (a) are modeled here as inversely related, i.e., *a* = 1–p.

^2^Kim et al. did examine the correlation between X and pre-B items, but not post-B items, as an index of AB prediction. The degree to which B features are re-activated when A was presented with an unexpected X instead, indicates how strongly B was predicted by A. This prediction strength was reported to significantly correlate with decrease in similarity between post-B and pre-A. While this finding was interpreted to uncover the underlying mechanism of differentiation, i.e., stronger violations leading to more differentiation of the violated item, such correlation would equally be predicted by the integration account, i.e., stronger violations leading to more integration of the violating item. In fact, the latter has been demonstrated empirically where larger prediction errors lead to better learning of the violating item (Greve et al., 2017).
